# Optimization of Ultrasound-Assisted Extraction via Sonotrode of Phenolic Compounds from Orange By-Products

**DOI:** 10.3390/foods10051120

**Published:** 2021-05-18

**Authors:** María del Carmen Razola-Díaz, Eduardo Jesús Guerra-Hernández, Celia Rodríguez-Pérez, Ana María Gómez-Caravaca, Belén García-Villanova, Vito Verardo

**Affiliations:** 1 Department of Nutrition and Food Science, Campus of Cartuja, University of Granada, 18011 Granada, Spain; carmenrazola@correo.ugr.es (M.d.C.R.-D.); ejguerra@ugr.es (E.J.G.-H.); belenv@ugr.es (B.G.-V.); 2 Department of Nutrition and Food Science, University of Granada, C/ Santander, 52005 Melilla, Spain; celiarp@ugr.es; 3 Biomedical Research Centre, Institute of Nutrition and Food Technology ‘José Mataix’, University of Granada, Avda del Conocimiento sn, 18100 Granada, Spain; anagomez@ugr.es; 4 Instituto de Investigación Biosanitaria ibs.GRANADA, 18071 Granada, Spain; 5 Department of Analytical Chemistry, University of Granada, Avda Fuentenueva, 18071 Granada, Spain

**Keywords:** Box–Behnken design, phenolic compounds, antioxidant activity, orange peel, sonotrode ultrasound-assisted extraction, HPLC-MS

## Abstract

Orange peel is the main by-product from orange juice industry. It is a known source of bioactive compounds, mostly phenolic compounds, and it has been widely studied for its healthy activities. Thus, this research focuses on the establishment of ultrasound-assisted extraction of phenolic compounds in orange peel using a sonotrode. For this purpose, a Box–Behnken design of 27 experiments was carried out with four independent factors—ratio ethanol/water (*v*/*v*), time (min), amplitude (%), and pulse (%). Quantitative analyses of phenolic compounds were performed and the antioxidant activity was measured by ABTS and DPPH methods. The validity of the experimental design was confirmed by ANOVA and the optimal sonotrode extraction conditions were obtained by response surface methodology (RSM). The extracts obtained in the established conditions were analyzed by High Performance Liquid Chromatography (HPLC) coupled to mass spectrometer detector and 74 polar compounds were identified. The highest phenolic content and antioxidant activity were obtained using 45/55 ethanol/water (*v*/*v*), 35 min, amplitude 90% (110 W), and pulse 100%. The established method allows an increment of phenolics recovery up to 60% higher than a conventional extraction. Moreover, the effect of drying on phenolic content was also evaluated.

## 1. Introduction

Orange is the second most produced fruit in the European Union, mainly in the countries of the Mediterranean basin where it supposes 6 million tons, almost 10% of the world production. Increasingly, citrus juices have gained great popularity, representing more than 50% of the juices available in international trade. From them, orange juice is the main product processed by the beverage industry and consumed throughout the world due to its high nutritional value and desirable sensory characteristics. Orange fruit processing to obtain juices or citrus-based beverages generates large amounts of waste by-products, such as peels, which are a rich source of soluble sugars, phenolic compounds, flavonoids, dietary fibers (cellulose, hemicelluloses, and pectin), vitamins, enzymes, and essential oils [1,2,3,4,5], which can be used for the production of nutritional dietary supplements [6]. Recently, several authors successfully used the orange by-products as ingredients with rheological and functional properties for the formulation of biscuits [7] and jams [8], and as fat replacer [9]. The orange peels have been reported to have antioxidant [10], anti-inflammatory [11], anti-cancer [12], anti-rheumatic [13], anti-diabetic [14], and cardioprotective activities [15], mainly attributed to its content in phenolic compounds. However, orange by-product, due to its high moisture content, is easily spoiled. By this way, the drying step is essential for decreasing the moisture content up to a level that inactivate the oxidant, enzymatic, and microbial degradation, ensuring a longer preservation among the time. It also could let to reduce the transport costs in an industrial level. Several drying techniques have been applied to the dehydration of citrus by-products, such as sun drying, hot air drying, freezing drying, microwave drying, etc. Between them, the best one referred to costs and yields and that can be scaled has been demonstrated to be oven-controlled drying [16]. After them, traditionally methods based on maceration and thermal extraction with different solvents such as hexane, acetone, methanol, etc., have been used to recover nutritionally valuable compounds from this type of fruit wastes [17]. According to this approach, the aim of this work is to establish the best ultrasound-assisted extraction via sonotrode to recover the phenolic compounds from orange by-products using food-grade solvents. The antioxidant activity of the extracts was also evaluated by DPPH (2,2-difenil-1-picrylhydrazyl) and ABTS (2,2′-Azino-bis (3-ethylbenzothiazoline-6-sulfonic acid) diammonium salt) assays. Moreover, the effect of drying on the phenolic composition was also evaluated. 

## 2. Materials and Methods

### 2.1. Chemicals and Samples

Gallic acid, DPPH and ABTS were purchased from Sigma-Aldrich (St. Louis, MO, USA). Na_2_CO_3_ was purchased from BDH AnalaR (Poole, England). Water was purified using a Milli-Q system (Millipore, Bedford, MA, USA). Vanillic acid, chlorogenic acid, ferulic acid, quercetin, and rutin were also acquired from Sigma-Aldrich (St. Louis, MO, USA). HPLC-grade water, Folin–Ciocalteu reagent and other reagents were purchased from Merck KGaA (Darmstadt, Germany).

Orange by-products (var. *Navelina*) were obtained after juice production. The resulting by-product was composed by the albedo, flavedo and rests of pulp of the orange with a humidity of 70 ± 1.5%. For the model the samples were dried at 60 °C according to Garau et al. [18]. Dried and fresh samples of by-products were grinded and frozen at −18 °C until the analyses.

### 2.2. Experimental Design

The conditions for obtaining the highest recovery of phenolic compounds and antioxidant activity from the orange by-products were optimized by using a Box–Behnken design and response surface methodology (RSM). The design was composed by 27 experiments structured in three blocks with three levels (−1, 0, +1). Each experiment was carried out in duplicate. The independent variables were ratio ethanol/water (0:100, 50:50, 100:0 *v*/*v*), time (5, 25, 45 min), amplitude (20%, 60%, 100%), and pulse (10%, 50%, 100%). The dependent variables were adjusted to a second order polynomial model equation (Equation (1)), where Υ represents the response variable, the total phenolic compounds (TPC) or the antioxidant assays ABTS or DPPH, X_i_ and X_j_ are the independent factors that affect the response, and β_0_, β_i_, β_ii_ and β_ij_ are the regression coefficients of the model (interception, linear, quadratic and interaction terms). Statistica 7.0 package (StatSoft, Tulsa, OK, USA) was used for the mathematical operations and simulations.

Equation (1). Second order polynomial equation.

(1) $$\Upsilon ={\;\beta }_{0}+\sum_{i=0}^{4}\beta_{i}X_{i}+\sum_{i=0}^{4}\beta_{\mathrm{ii}}X_{\mathrm{ii}}^{2}+\sum_{i=0}^{4}\sum_{j=0}^{4}\beta_{\mathrm{ii}}X_{i}X_{j}$$

ANOVA assay was performed in order to evaluate the adjustment of the models having into account the regression coefficients, the *p*-values of the regressions and the lacks of fit. The optimum conditions were established using RSM.

### 2.3. Extraction of Phenolic Compounds from Orange By-Products by Sonotrode Ultrasonic Extraction

Orange by-products (0.5 g) were extracted with an ethanol/water solution (*v*/*v*) (100 mL) by a sonotrode (UP400St ultrasonic processor, Hielscher, Germany) according to the parameters showed in Table 1. After the extraction, the samples were centrifuged at 3500 rpm for 15 min and the supernatant was stored at −18 °C until the analyses.

### 2.4. Conventional Extraction of Phenolic Compounds from Orange By-Products

The conventional extraction was carried out according to Abdurrahman et al. [19] with slightly modifications. Briefly, 0.5 g of orange by-products was added of 100 mL of ethanol/water (45/55 *v*/*v*) and it was agitated by a magnetic stirrer during 35 min at 35 °C. The samples were centrifuged at 3500 rpm for 15 min and the supernatant were collected and stored at −18 °C until the analyses.

### 2.5. Determination of Total Phenolic Compounds by Folin–Ciocalteu Assay

Total phenolic compounds were determined by Folin–Ciocalteu spectrophotometric method [20]. Thus, 100 µL of the extract was added of 500 µL of Folin–Ciocalteu reagent and 6 mL of MilliQ water. The flask was agitated for a minute. After that, 2 mL of 15% (*w*/*v*) Na_2_CO_3_ was added and made up to 10 mL with MilliQ water, and stored at dark conditions. The measures were carried out after 2 h at 750 nm and 25 °C with a UV–Visible spectrophotometer (Spectrophotometer 300 Array, UV–Vis, single beam, Shimadzu, Duisburg, Germany). The results were compared to a standard curve of gallic acid (1, 5, 10, 25, 50, 100, 250 ppm) to calculate the TPC. Results are expressed as mg gallic acid equivalents (GAE)/g dry weight (d.w.).

### 2.6. Determination of Phenolic Compounds from Orange By-Products by HPLC-ESI-TOF-MS

The analyses of the orange by-products using the optimized conditions obtained with the Box–Behnken design were carried out in duplicate on an ACQUITY Ultra Performance LC system (Waters Corporation, Milford, MA, USA) coupled to an electrospray ionization (ESI) source operating in the negative mode and a time-of-flight (TOF) mass detector (Waters Corporation, Milford, MA, USA). The compounds of interest were separated on an ACQUITY UPLC BEH Shield RP18 column (1.7 µm, 2.1 mm × 100 mm; Waters Corporation, Milford, MA, USA) at 40 °C using a gradient previously stated by Verni et al. [21] using water containing 1% acetic acid as mobile phase A and acetonitrile as mobile phase B. The data were elaborated using MassLynx 4.1 software (Waters Corporation, Milford, MA, USA).

### 2.7. Antioxidant Assays DPPH and ABTS

The antioxidant capacity was evaluated in the 27 orange by-products extracts by two different methods. The first one was the developed by Re at al. [22] in which the monocation ABTS^•+^ is generated by oxidation of the ABTS with potassium persulfate in the dark at room temperature for 12–24 h. For each extract, it was added 1 mL of this ABTS solution to 0.01 mL of extract and it was measured the detriment of absorbance during 10 min at 734 nm. The results were compared with a standard curve of Trolox (1, 10, 20, 50, 80, 100, 150, 200 ppm). DPPH radical scavenging activity was assayed with a method proposed by several authors [23,24]. Then, 100 µL of each extract was added of 2.9 mL of DPPH, and after rapid stirring, the bleaching power of the extract was observed in a time interval from 0 to 30 min at 517 nm. The results were compared with a standard curve of Trolox in methanol/water (4:1, *v*/*v*) (1, 10, 20, 50, 80, 100, 150, 200 ppm). Results for both assays are expressed as mg Trolox equivalents (TE)/g d.w.

## 3. Results and Discussion

### 3.1. Determination of Total Phenolic Compounds and Antioxidant Capacity in Orange By-Products

The Box–Behnken model coupled to response surface methodology was used to optimize the extraction of the extract with the highest TPC and the highest antioxidant capacity (Table 1).

For TPC, the observed values ranged between 8.7 and 29.7 mg GAE/g d.w. The lowest value corresponds to the conditions 100% ethanol, 25 min, 20% amplitude, and 50% pulse, and the highest one to ethanol/water (50: 50, *v*/*v*), 25 min, 100% amplitude, and 100% pulse.

For the antioxidant assays, values ranged from 9.5 to 26.4 mg TE/g d.w. with the DPPH technique, and from 11.5–40.5 mg TE/g d.w. with the ABTS one. The minimum value with both methods was obtained using only ethanol (100%), whereas the highest ones were observed by using a mixture of 50% ethanol and 50% water, similarly to the results described for TPC.

### 3.2. Fitting the Model

The experimental data was analyzed adjusting it to a second order polynomial equation, a regression model that, using the least squares method, provides the lowest residual value (Equation (1)). All the regression coefficients are shown in Table 2.

The non-significant terms with a significance level of *p* < 0.05 were discarded, and the model was recalculated only with significant terms. For the three response variables, the individual linear factors ratio ethanol/water (β_1_), time (β_2_) and pulse (β_4_) showed significant effects. For the TPC and DPPH, the crossed interaction β_12_ was significant, and the crossed interactions β_23_ and β_34_ were significant for TPC and ABTS. Moreover, the quadratic term β_11_ was significant for the three response variables; meanwhile, β_22_ was significant for ABTS and DPPH, and β_44_ only for TPC. The amplitude (β_3_) and the rest of interactions did not have significant effect in the meanings chosen. In addition, for the three responses it was revealed a high correlation between the factors and the response variable (R^2^ = 0.9564, 0.9286, and 0.8694 for TPC, ABTS and DPPH, respectively). The validity of the model was tested with ANOVA, which showed that, the models can be statistically accepted because the regression model *p* values are lower than 0.05 and the lacks of fit for the three variables are non-significant (*p* > 0.05).

### 3.3. Confirmation of the Optimal Extraction Parameters by Sonotrode

**Figure 1:**
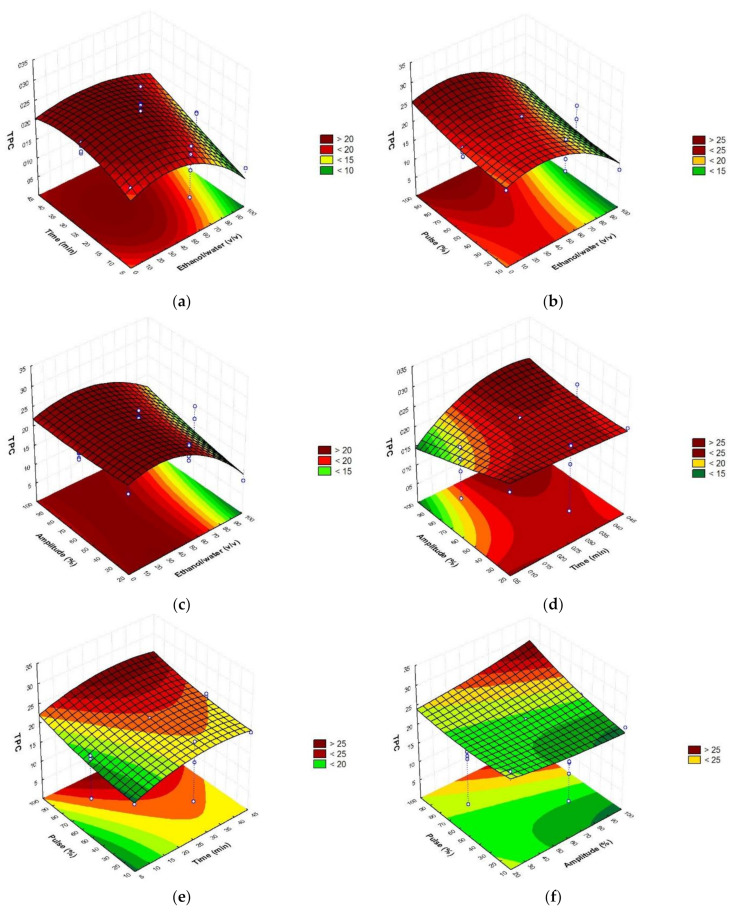
Response surface graphs (**a**–**f**) showing the combined effects of the process variables: Ethanol/water (*v*/*v*), time (min), amplitude (%), and pulse (%), for TPC (mg GAE/g d.w.).

**Figure 2:**
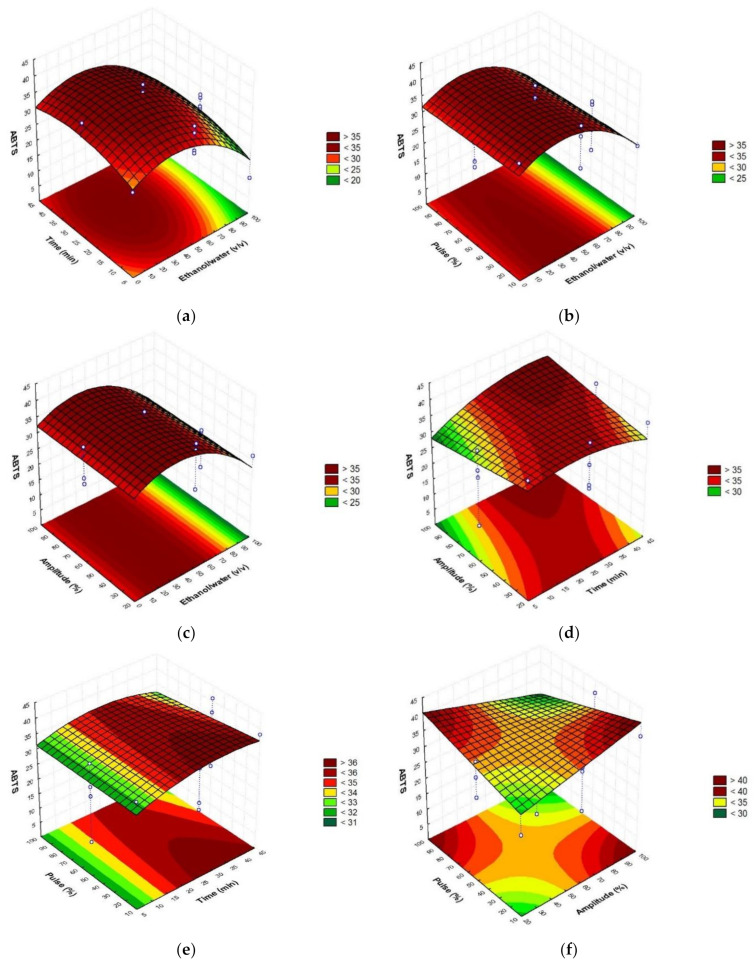
Response surface graphs (**a**–**f**) showing the combined effects of the process variables: Ethanol/water (*v*/*v*), time (min), amplitude (%), and pulse (%), for ABTS antioxidant assay (mg TE/g d.w.).

**Figure 3:**
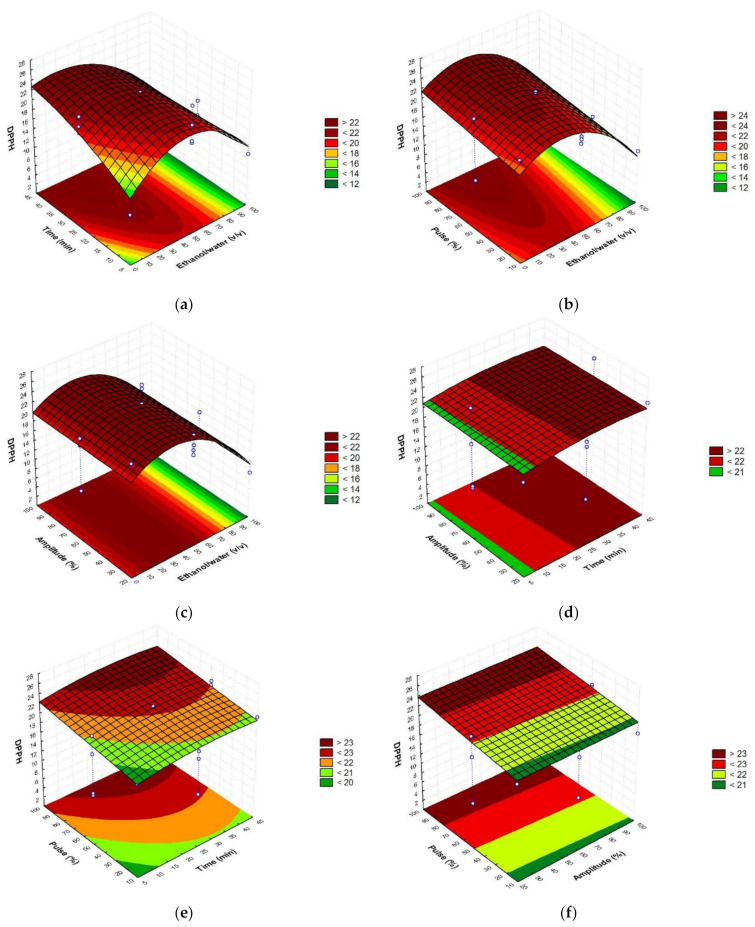
Response surface graphs (**a**–**f**) showing the combined effects of the process variables: Ethanol/water (*v*/*v*), time (min), amplitude (%), and pulse (%), for DPPH antioxidant assay (mg TE/g d.w.).

To establish the optimal conditions of extraction, the obtained response surfaces (Figure 1, Figure 2 and Figure 3) were studied. 

As can be seen in the figures, the major content of phenolic compounds with the highest antioxidant activity could be obtained with the highest amplitude and pulse values and the intermedium ethanol/water ratio and time values.

Briefly, optimal sonotrode extraction conditions were 45% ethanol/water (*v*/*v*), 35 min, amplitude 90% (110 W), and pulse 100% (Table 3). Verifying the accuracy of the mathematical model, the obtained values did not report significant differences with the predicted values, showing coefficients of variation <1 in the case of the total phenolic compounds, and the antioxidant assays (DPPH and ABTS). According to the results, ethanol was not an efficient solvent when used pure, showing better results when it was combined with water in a proportion equal or minor to 50%. This occurs due to the increased solvation provided by the presence of water. Additionally, the optimum time was chosen as lower as possible in order to develop a quick procedure. The highest values of amplitude and pulse are necessary in order to reach higher powers and finally obtain better performances.

**Figure 4:**
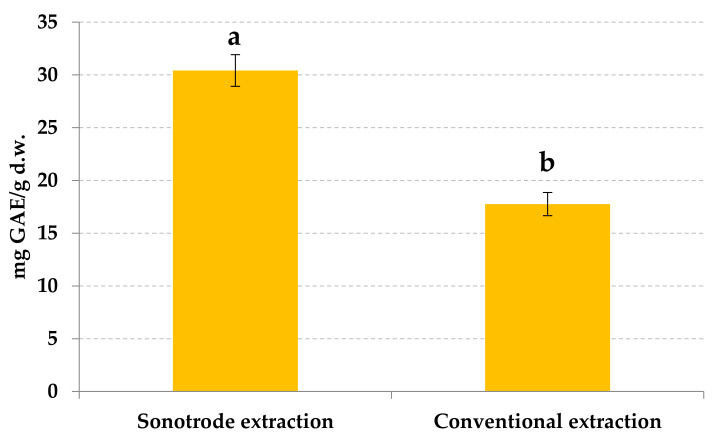
Comparison of phenolic content in orange by-products extracts obtained by sonotrode and conventional extraction. Different letters reported statistical significant differences.

The value of TPC obtained by sonotrode was compared with the same values of the extract obtained by the conventional extraction. The recovery of phenolic compounds by the proposed extraction using sonotrode was 60% higher than obtained with the conventional one (Figure 4).

Victor et al. [25] and Lagha-Benamrouche et al. [26] reached similar results than the reported in this study but using higher times of extraction. Other authors optimized the phenolic extraction from the orange peel by other methods as high voltage electrical discharges [6], microwave-assisted extraction [27], and pressurized liquid extraction [3], but they noticed lower yields compared to our results (Table 4). The ultrasound-assisted extraction of phenolic compounds from orange peel using ultrasonic bath has been performed by several authors using ethanol [3,28,29] or acetone [27], obtaining extraction times quite similar to our optimum one. In addition, some pre-treatments have been performed by other authors in orange by-products for enhancing the extraction of phenolic compounds. On the one hand, Shahram et al. [30] improved the usual ultrasonic bath extraction combining it with an enzymatic process. However, the TPC seemed to be lower than the previous studies without enzymes. Overall, it required longer times because of the lower pH. In addition, pectinases are vegetal cells, and the ultrasonic waves can damage them, making them to lose their potential effect. In spite of the fact that they did not achieve a high yield, it could be had into account for future studies making slightly modifications. On the other hand, Luengo et al. [31] studied for the first time the use of pulsed electric fields as pretreatment, obtaining an increment of 23% in the yield in front of the samples directly submitted to pressing extraction. Another interesting pre-treatment was the one carried out in orange peel by El-Kantar et al. [32], who achieved an improvement of extraction about 47% using infrared radiation previously to the conventional water extraction. Although the final results obtained were not satisfactory, these two pre-treatments also ought to be had into account for future studies combining with this optimized sonotrode-assisted extraction.

Bringing into account the antioxidant assays, the optimal results obtained were 26.37 ± 1.6 and 35.62 ± 2.1 mg TE/g d.w. for the DPPH and ABTS, respectively. DPPH technique has been mostly used in bibliography in this kind of matrix. Comparing results, the optimal value obtained for DPPH is higher than those obtained by Hernández-Carranza et al. [33] (8.94 mg TE/g d.w.) and Barrales et al. [3] (4.30 mg TE/g d.w.). However, Lachos-Pérez et al. [35] got an optimal result slightly higher (26.78 mg TE/g d.w.) but with a time of extraction of 2 h, much higher that the optimized value obtained in this study.

### 3.4. Analytical Parameters of the Method

Five calibration curves were made in order to quantify the phenolic compounds identified in the orange by-product. The curves were made of vanillic acid, chlorogenic acid, ferulic acid, quercetin, and rutin. In Table 5 are contained the standards used, the calibration ranges and curves, the regression coefficients, and the limits of detection (LOD) and of quantification (LOQ).

The calibration curves were elaborated by using the peak areas of each standard measured by HPLC at different concentrations. Calibration ranges were determined previously according to the LOQ values. The regression coefficients were >0.99 in all cases which means that all calibration curves had good linearity. LOD ranged between 0.04 and 0.47 µg/mL, and LOQ between 0.14 and 1.57 µg/mL.

### 3.5. Identification of Phenolic Compounds by HPLC-ESI-TOF-MS

The optimal conditions of extractions were applied to extract the phenolic compounds from fresh and dried orange by-products in order to evaluate the effect of the drying step. The extracts were characterized by HPLC-MS and a total of seventy-four compounds were identified; forty-four were flavonoids, fourteen phenolic acids, fourteen terpenoids, and two other polar compounds.

All the identified compounds are described in Table 6 with their retention time, molecular formula, experimental and calculated *m/z*, score and error (ppm). For ensuring the mass accuracy, the tolerances chosen had a score higher than 85% and error lower than 5 ppm. To identify the compounds the generated molecular formula and some in source fragments were checked and studied comparing also with different databases such as PUBCHEM, Phenol-Explorer and literature.

*Phenolic acids*. Thirteen hydroxycinnamic acids and one hydroxybenzoic acid have been found.

Five caffeic acid derivates (peaks 1, 7, 13, 14, and 23) have been detected in this study. Two of them, at *m*/*z* 251 (peaks 1 and 3), have been tentatively identified as caffeoylglycolic acid methyl esters isomers a and b. This compound was first reported in leaves of *Parthenocissus tricuspidate* by Saleem et al. [36]. Another two derivatives at *m/z* 295 are named as caffeoylmalic acids isomers corresponding to peaks 14 and 23 described previously in plants by Hahn et al. [37], being the major source the lettuce. Moreover, the last derivate of caffeic acid that correspond to the compound found at peak 7 has been assigned to caffeic acid 3-*O*-glucuronide according to Tang et al. [38], who found the compound in hops extracts. To our knowledge, there are not previous references of these compounds found in citrus neither in orange matrixes; however, caffeic acid is a well-known compound found in orange peel by some authors [39,40].

Peaks 5, 6, and 10, with molecular formula C_16_H_18_O_11_, were identified as 2-(E)-*O*-feruloyl-D-galactaric acids isomers a, b and c first identified in orange peel extracts by Risch et al. [41].

Derivatives of ferulic and sinapic acids have been identified in the peaks 9 and 12, tentatively named as ferulic acid *O*-glucoside and sinapic acid *O*-glucoside, respectively. It seems to be in concordance with Lu et al. [42] who identified in orange pulp a mixture of ferulic acid and sinapic acid glucosides. In addition, both acids have been previously described in orange peel by other authors [27], but not these glucoside derivatives.

At time 6.545 (peak 15) another hydroxycinnamic acid has been tentatively identified as a sinapinic acid derivate according with Anagnostopoulou et al. [43] and Bocco et al. [44] namely as sinapinic acid-*O*-glucuronide described in orange peels.

The compounds found at peaks 11 and 16 with molecular formula C_16_H_16_O_10_, have been identified as feruloyl isocitric acid isomers, described previously in plants by Masike et al. [45], and never described before in orange peel extracts. However, isocitric acid has been previously found in citrus peel matrices [46]. Furthermore, the ratio citric/isocitric acids usually is used for quality control in the orange juice; in fact, a ratio higher than 130 usually suggests that the beverage has been correcting by adding citric acid [47].

Finally, a hydroxybenzoic acid has been identified at time 3.806 min (peak 2). According with Nazir et al. [48] it was tentatively named as norbergenin. They identified this compound from *Bergenia stracheyi*, but there are no references in citrus matrices.

*Flavonoids.* As a majoritarian group, forty-four flavonoids have been identified in the orange peel.

Peaks 28, 37, 42, 51, 52 and 62 were assigned to flavanone compounds corresponding to eriocitrin, narirutin, hesperidin, neohesperidin, didymin, and naringenin, respectively. Moreover, rutin isomers have been found at *m/z* 609 (peaks 17 and 40). These compounds are in agreement with several authors who identified them in orange peel [39,49,50,51,52,53,54,55,56].

With a molecular formula C_21_H_22_O_10_ (peak 20), the flavonoid prunin has been identified in concordance with Berhow et al. [57] and Castillo et al. [58], who found it for the first times as a precursor of neohesperidin and naringin in citrus matrices.

According with Oboh et al. [54] and Omoba et al. [56] the compound found at time 8.676 min (peak 27) was identified as quercitrin, found in orange’s juices and peels, respectively.

Two flavonoid isomers with *m/z* 563 and molecular formula C_26_H_28_O_14_ have been found (peaks 30 and 33) and they were assigned to vitexin-*O*-pentoside according to Fayek et al. [59] who found this compound in *Citrus reticulata* peel. It also could be an apigenin derivate called apigenin 6-*C*-glucoside 8-*C*-arabinoside in concordance with Lu et al. [42] who found a similar compound (apigenin-6,8-di-*C*-glucoside) in an orange variety.

At retention time 16.524 min (peak 73), and according with Gattuso et al. [60], it has been identified the flavonoid as isosakuranetin, previously found in orange juice. This compound has been found for the first time in *Citrus reticulata* peel by Fayek et al. [59], and they named it as dihydroxy-methoxyflavanone, so it is the first time this compound is identified in orange by-products. 

Two nobiletin derivates has been found at times 13.88 and 16.57 min (peaks 60 and 74). According with Goh et al. [61], they were tentatively identified as demethylnobiletin and 3′,4′-didemethylnobiletin, respectively, described in the orange peel.

A hesperidin derivate, alpha-glucosyl hesperidin, previously found in the orange peel by several authors [62,63,64,65,66] has been identified at time 8.481 min (peak 25). In addition, a naringin derivate found at peak 34 was assigned to naringin hydrate according with Iglesias-Carres et al. [55] and Fayek et al. [59].

Isorhamnetin-3-*O*-rutinoside isomers were identified (peaks 21, 22 and 48) with *m*/*z* 623. At 7.194 min (peak 18), the detected signal was identified as dihydroisorhamnetin-7-rutinoside. In addition, the compounds found at times 12.693 and 13.814 min (peaks 54 and 59), were identified as apigenin-7-*O*-neohesperidoside and quercetin-3-*O*-rutinoside-7-*O*-glucoside, respectively. The present data is in agreement with Abad-García et al. [67] who identified fifty-eight new compounds in citrus juices.

Compounds 19, 31, 35, 36, and 47 presented the same mass (593) and molecular formula (C_27_H_30_O_15_), and could be identified as apigenin-di-*C*-hexoside (Vicenin-2) according with Fayek et al. [59] who found this compound in *Citrus sinensis* peel and identified it according with Brito et al. [68], who previously detected vicenin-2 in *Citrus aurantiifolia*.

The compounds with molecular formula C_26_H_28_O_15_ (peaks 24 and 26) can be identified as luteolin-*C*-hexoside-*C*-pentoside isomers characterized for the first time by Fayek et al. [59] in *Citrus sinensis* peel according to Roriz et al. [69] who described the compound in *Cymbopogon citratus*.

Six kaempferol derivates were found. The first one kaempferol derivate with a formula C_39_H_32_O_13_ (peak 43), was tentatively named as kaempferol 3-*O*-[3″,6″-di-*O*-(E)-cinnamoyl]-*β*-d-glucopyranoside. The second one with m/z 813 (peaks 45 and 46) was tentatively named as kaempferol-3-[2″-glucosyl-6″-acetyl-galactoside]-7-glucoside. Previously, this compound had been detected only in herbs and spices, but not quantified [70]. The third and fourth ones (peaks 57 and 58) with m/z 635.1617 and 709.1987 are kaempferol 3-*O*-(6″-*O*-acetyl) glucoside-7-*O*-rhamnoside, and kaempferol 3-apiosyl-(1->4)-rhamnoside-7-rhamnoside, respectively. The fifth derivate is kaempferol-3-*O*-sinapoyl-caffeoyl-sophoroside-7-*O*-glucoside with molecular formula C_53_H_56_O_28_ (peaks 63 and 64 respectively). The sixth one was found at time 14.786 min (peak 67) and is kaempferol-3-*O*-feruloyl-caffeoyl-sophoroside-7-*O*-glucoside. All this compounds have been tentatively identified based on other kaempferol derivates found in other plant sources [71,72]. Although kaempferol derivates are known to be present in citrus peels, no previous references have been found, neither in orange peel.

With a mass (*m/z*) 443 (peak 4), this flavonoid has never been described in the orange peel, nor in the citrus family so far. However, according to Zanzer et al. [73], this compound was characterized before in black pepper, so it has been tentatively identified as cynaroside A.

The flavonoid with formula C_27_H_30_O_17_ (peak 32) was tentatively identified as quercetin-*O*-dihexoside as Fayek et al. [59] found it previously in *Citrus reticulata* and *Citrus sinensis* peels.

With a mass of 651, at 11.613 min (peak 49) the flavonoid found can be named as kaempferol-dihexosyl acetate according to Fayek et al. [59], who recently found it from *Citrus aurantiifolia* peel for the first time.

At time 10.074 min and with a mass of 561 (peak 38), a controversial compound was identified. According with Fayek et al. [59], this compound could be an unknown flavonoid, as they found it in *Citrus paradisi* peel. Although no other references have been found in citrus matrixes, for the context this affirmation can be accepted.

The compound naringin 6″-malonate (peak 56) was tentatively identified according with Berhow et al. [74] who found this compound and other malonic acid ester derivatives of naringin for the first time in grapefruit. Kanes et al. [75] found this compound in some rutaceae citrus species, but no references has been found related to orange peel.

With a molecular formula C_17_H_14_O_6_ (peak 71) this compound has been assigned to pectolinarigenin according with Cheriet et al. [76] who described it in several plants and fruits as oranges. However, this compound has been previously named in *Citrus sinensis* peel by Fayek et al. [59] as dihydroxy-dimethoxyflavone, previously identified in *Citrus reticulata* peel by Yang et al. [77].

*Terpenoids*. Talking about the terpenoids, 13 limonoids have been identified. They have been identified according with Gualdani et al. [78] and Shi et al. [79], who characterized limonoids from several citrus species and matrixes including orange peel. The compounds found are named limonin 17-*β*-d-glucopyranoside, 6-keto-7-*β*-deacetylnomilol 17-*O*-*β*-d-glucopyranoside, deacetylnomilinic acid-17-*β*-d-glucoside, isoobacunoic acid 17-*β*-d-glucoside, nomilin 17-*O*-*β*-d-glucopyranoside, nomilinic acid 17-*β*-d-glucoside, obacunone 17-*β*-d-glucoside, deacetylnomilin acid, limonol, epilimonin, limonin, nomilinic acid, and deoxylimonin (peaks 29, 39, 41, 44, 50, 53, 55 65, 66, 68, 69, 70 and 72, respectively). Another terpene different from those, a sesquiterpene, has been found at 5.7 min (peak 8). According to Umehara et al. [80], it was tentatively identified as citroside. They found this compound from leaves of *Citrus unshiu*.

*Other polar compounds*. Finally, other two polar compounds have been found in the orange by-product. The ion found at *m*/*z* 191.0198 (peak 3) at 4.278 min, is well known as citric acid, and according to Liew et al. [81], it has been previously identified in orange peel extracts. Meanwhile the other compound (peak 61) with the molecular formula C_35_H_40_O_20_ was only found match with the compound methyl 2-[(2S,4R,5S,6R)-4-acetyloxy-6-(acetyloxymethyl)-5-[(2R,4R,5S,6R)-4,5-diacetyloxy-6-(acetyloxymethyl) oxan-2-yl] oxyoxan-2-yl] oxy-3,4,5-trihydroxy-6-oxobenzo [7] annulene-8-carboxylate that no has previous references.

### 3.6. Quantification of Phenolic Compounds in Orange Peel Extracts

A total of 14 phenolic acids and 44 flavonoids were quantified in orange by-product optimal extracts (Table 7).

Phenolic acids represented the 61% of the total phenolic compounds found in the dry orange by-product being 48% of those found in the fresh by-product. The most concentrated phenolic acid in fresh and dry by-products were 2-(E)-*O*-feruloyl-d-galactaric acid isomers b and c, accounting for 18.6 and 19.5%, respectively. 2-(E)-*O*-feruloyl-D-galactaric acid isomers were approximately, 40–50% of the phenolic acids present in orange by-products. After those compounds, norbergenin accounted for 12% of the phenolic acids in both by-products. The major caffeic acid derivatives were caffeoylglycolic acid methyl ester isomers a and b, accounting for the 18% and 16% in the fresh and dry by-products, respectively.

The total amount of flavonoids found in the dry and fresh samples represented the 39% and 52% of the total phenolic compounds, respectively. The two most concentrated flavonoids, both, in the fresh and dry by-products were hesperidin and narirutin. However, in the fresh sample, they account for 45% of the present flavonoids, whereas in the dry one they were the 35%. A study which optimized the extraction of these two compounds from the orange peel by subcritical water extraction, obtained an extract in which both compounds were approximately 21% of the total amount of flavonoids, a smaller value than the obtained with this optimized technique [35]. Other studies agree with us about the major compound found, hesperidin. Using an ultrasonic bath, Khan et al. found hesperidin as major compound getting an amount of 146.73 µg/g d.w. [29]. Barrales et al. obtained an extract mainly composed by hesperidin (160 µg/g d.w.) and narirutin (120 µg/g d.w.) [3] with ultrasound bath technology. Despite not being the principal aim of this study, hesperidin and narirutin content was higher than the described in previous studies that used ultrasound technology applied to orange by-products.

The fourth most abundant flavonoid was apigenin-di-*C*-hexoside (Vicenin-2) isomer a, being around 17.5% of the flavonoids in both samples. For the dry by-product the fifth and sixth most abundant compounds were an unknown flavonoid (6.8%) and vitexin-O-pentoside isomer b (5.1%), whereas for the fresh one they are didymin (4.5%), and prunin (3.8%).

**Figure 5:**
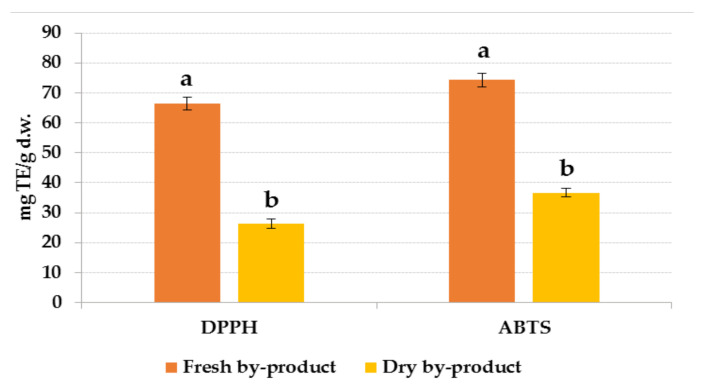
Comparison of antioxidant activities by two methods (DPPH and ABTS) in fresh and dry orange by-products extracts expressed in mg TE/g d.w. Different letters reported statistical significant differences.

Taking into account the antioxidant activity, the fresh orange by-product has been found to have a higher activity than the dry one, with values of 66.39 and 74.32 mg TE/g d.w. for the DPPH and ABTS assays, respectively (Figure 5).

This can be explained because the fresh by-product has a higher amount of total phenolic compounds than the dry one. The loss is around 25% of the total phenolic compounds which means the loss of 43% of the flavonoids, 6% of the phenolic acids and consequently the loss of 50–60% of the antioxidant activity.

## 4. Conclusions

An extraction based on ultrasound-assisted extraction by sonotrode has been optimized for obtaining the higher amounts of phenolic compounds with high antioxidant activity from the orange by-products. The results of this study have shown that applying the sonotrode extraction is possible to increase 60% more the phenolic recovery compared to a conventional extraction procedure as maceration. Moreover, sonotrode extraction has demonstrated to be faster than the conventional ones. In addition, the extracts have been exhaustively characterized identifying a total of 74 compounds in orange peels being 32 of them tentatively identified in orange by-products for the first time. The distribution of the phenolic compounds is mainly phenolic acids and flavonoids, especially flavonoid glycosides. The effect of drying was also evaluated and, as expected, it caused the loss of 25% of phenolic compounds; perhaps, an optimization of the drying conditions should be carried out in order to limit the phenolic degradation. Finally, the sonotrode ultrasound technology could be scaled-up to pilot and industrial scale in order to obtain orange by-products extracts that could be used as functional ingredients for food, feed and cosmeceutical scopes. However, to corroborate the potential (bio)-activity of the extracts, further in vitro and in vivo assays should be done.

## Figures and Tables

**Table 1:** Box–Behnken design with natural and coded values (parenthesis) of the conditions of extraction and the experimental results obtained for TPC, and antioxidant assays (ABTS and DPPH) expressed with the average and the standard deviation.

	Independent Factors				Dependent Factors		
	X1	X2	X3	X4	TPC (mg GAE/g d.w.)	ABTS (mg TE/g d.w.)	DPPH (mg TE/g d.w.)
1	0 (−1)	5 (−1)	60 (0)	50 (0)	20.46 ± 0.18 ^e–g^	26.67 ± 0.39 ^e,f^	10.55 ± 0.03 ^b^
2	100 (1)	5 (−1)	60 (0)	50 (0)	10.33 ± 0.10 ^b^	11.56 ± 0.26 ^a^	10.94 ± 0.21 ^c^
3	0 (−1)	45 (1)	60 (0)	50 (0)	21.64 ± 0.21 ^f–i^	24.92 ± 0.06 ^d,e^	19.79 ± 0.04 ^i^
4	100 (1)	45 (1)	60 (0)	50 (0)	19.55 ± 0.35 ^e^	18.28 ± 0.38 ^b^	11.07 ± 0.06 ^c^
5	50 (0)	25 (0)	20 (−1)	10 (−1)	25.33 ± 0.14 ^l^	25.21 ± 0.26 ^d,e^	20.02 ± 0.06 ^i^
6	50 (0)	25 (0)	100 (1)	10 (−1)	21.95 ± 0.16 ^g–j^	36.62 ± 0.50 ^n–p^	18.54 ± 0.05 ^f^
7	50 (0)	25 (0)	20 (−1)	100 (1)	25.12 ± 0.35 ^l^	39.13 ± 0.79 ^o–q^	21.05 ± 0.04 ^k,l^
8	50 (0)	25 (0)	100 (1)	100 (1)	29.75 ± 0.36 ^m^	30.14 ± 0.01 ^h, i^	20.95 ± 0.05 ^k^
9	50 (0)	25 (0)	60 (0)	50 (0)	23.59 ± 0.92 ^j,k^	34.97 ± 0.13 ^l, m^	22.43 ± 0.06 ^m,n^
10	0 (−1)	25 (0)	60 (0)	10 (−1)	20.08 ± 0.35 ^e,f^	36.27 ± 0.26 ^l–n^	21.21 ± 0.05 ^k,l^
11	100 (1)	25 (0)	60 (0)	10 (−1)	10.11 ± 0.37 ^b^	22.87 ± 0.51 ^c^	11.16 ± 0.04 ^c^
12	0 (−1)	25 (0)	60 (0)	100 (1)	21.95 ± 0.42 ^h–k^	33.07 ± 0.78 ^j,k^	19.93 ± 0.05 ^i^
13	100 (1)	25 (0)	60 (0)	100 (1)	16.27 ± 0.35 ^c,d^	23.60 ± 0.77 ^c,d^	15.44 ± 0.05 ^e^
14	50 (0)	5 (−1)	20 (−1)	50 (0)	21.30 ± 0.03 ^f–h^	37.18 ± 0.77 ^m–o^	19.03 ± 0.05 ^g^
15	50 (0)	45 (1)	20 (−1)	50 (0)	22.40 ± 0.14 ^h–k^	36.28 ± 0.64 ^m–p^	23.07 ± 0.05 ^p^
16	50 (0)	5 (−1)	100 (1)	50 (0)	10.31 ± 0.04 ^b^	29.71 ± 0.27 ^g, h^	22.69 ± 0.06 ^o^
17	50 (0)	45 (1)	100 (1)	50 (0)	23.03 ± 0.14 ^j,k^	40.92 ± 0.64 ^q^	22.23 ±0.05 ^m^
18	50 (0)	25 (0)	60 (0)	50 (0)	22.35 ± 0.07 ^h–k^	34.92 ± 0.59 ^k,l^	21.20 ± 0.08 ^l^
19	0 (−1)	25 (0)	20 (−1)	50 (0)	20.56 ± 0.35 ^e–g^	32.53 ± 0.37 ^j,k^	23.19 ± 0.06 ^p^
20	100 (1)	25 (0)	20 (−1)	50 (0)	8.66 ± 0.06 ^a^	26.33 ± 0.12 ^e,f^	9.54 ± 0.04 ^a^
21	0 (−1)	25 (0)	100 (1)	50 (0)	22.81 ± 0.26 ^j,k^	32.51 ± 0.03 ^j,k^	20.29 ± 0.05 ^j^
22	100 (1)	25 (0)	100 (1)	50 (0)	15.98 ± 0.21 ^c^	27.35 ± 0.96 ^f,g^	12.30 ± 0.04 ^d^
23	50 (0)	5 (−1)	60 (0)	10 (−1)	17.30 ± 0.42 ^d^	35.04 ± 0.19 ^l–n^	19.41 ± 0.04 ^h^
24	50 (0)	45 (1)	60 (0)	10 (−1)	20.42 ± 0.09 ^e–g^	38.15 ± 0.76 ^p,q^	21.28 ± 0.04 ^l^
25	50 (0)	5 (−1)	60 (0)	100 (1)	23.37 ± 0.13 ^k^	28.93 ± 0.15 ^g, h^	26.45 ± 0.04 ^r^
26	50 (0)	45 (1)	60 (0)	100 (1)	25.30 ± 0.08 ^l^	31.37 ± 0.37 ^i,j^	25.65 ± 0.04 ^q^
27	50 (0)	25 (0)	60 (0)	50 (0)	22.52 ± 0.36 ^i–k^	32.62 ± 0.27 ^j,k^	22.61 ± 0.01 ^n,o^

X_1–4_: Ethanol/water (*v*/*v*), time (min), amplitude (%), pulse (%). TPC: Total phenolic compounds. GAE: Gallic acid equivalents. TE: Trolox equivalents. d.w.: dry weight. Different letters in the same column showed significant differences.

**Table 2:** Estimated regression coefficients of the adjusted second-order polynomial equation and analysis of variance (ANOVA) of the model.

Regression Coefficients	Response					
	TPC		ABTS		DPPH	
	Effect	*p* Value	Effect	*p* Value	Effect	*p* Value
β_0_	20.9165 *	0.0000	25.5081 *	0.0000	16.4511 *	0.0001
Lineal						
β_1_	−7.39159 *	0.0000	−6.9354 *	0.000	−7.1540 *	0.0036
β_2_	7.14405 *	0.0001	4.1242 *	0.0189	3.9375 *	0.0343
β_3_	0.1177	0.8784	−2.5229	0.6391	1.2579	0.7225
β_4_	5.0592 *	0.0002	−6.3625 *	0.0227	4.2319 *	0.0215
Crossed						
β_12_	4.0196 *	0.0211	4.2385	0.1091	−4.5509 *	0.0274
β_13_	2.5322	0.0637	0.5152	0.7383	2.8321	0.0665
β_14_	2.1488	0.0855	1.9652	0.2811	2.7874	0.0684
β_23_	5.8083 *	0.0023	6.0598 *	0.0182	−2.2457	0.4190
β_24_	−0.5977	0.4677	−0.3305	0.8286	−1.3348	0.2248
β_34_	4.0099 *	0.0214	−10.1977 *	0.0005	0.6917	0.4634
Quadratic						
β_11_	1.9745 *	0.0001	12.6447 *	0.0000	8.0299 *	0.0020
β_22_	−1.0824	0.1228	7.1362 *	0.0000	2.1692 *	0.03400
β_33_	−1.6717	0.0569	0.7439	0.4660	1.1751	0.1326
β_44_	−2.2687 *	0.0336	1.9455	0.1091	0.4520	0.3771
R^2^	0.9564		0.9286		0.8694	
*p* Model	0.0011		0.0043		0.0001	
*p* Lack of fit	0.1522		0.2624		0.1117	

* Significant at α ≤ 0.05; 1 Ethanol/water ratio (*v*/*v*), 2 time, 3 amplitude, 4 pulse.

**Table 3:** Optimal conditions selected and the model predicted values with the obtained values expressed with the mean and the standard deviation.

Parameter	Optimal Conditions		
Ethanol/water (*v*/*v*)	45		
Time (min)	35		
Amplitude (%)	90		
Pulse (%)	100		
	TPC	DPPH	ABTS
Predicted value (mg/g d.w.)	29.36 ± 3.5	24.44 ± 3.6	32.02 ± 7.0
Obtained value (mg/g d.w.)	30.42 ± 1.5	26.37 ± 1.6	35.62 ± 2.1
Coefficient of variation	0.025	0.053	0.075

**Table 4:** Previous research about other technologies used for extracting phenolic compounds from the orange by-products with the conditions used and the total phenolic compounds (TPC) obtained.

Technology Used	Conditions	TPC (mg GAE/g d.w.)	Reference
Solvent extraction	Water, 60 °C, 12 h	6.89	[33]
	Methanol, 55 °C, 3 h	28.00	[25]
	Methanol/water 80:20, 20 °C, 22 h	25.60	[26]
	Methanol/sample 20:1 (*v*/*w*), 25 °C, 72 h	18.50	[34]
	Acetone/sample 20:1 (*v*/*w*), 25 °C, 72 h	18.00	[34]
	Acetone 50%/sample 50:1 (*v*/*w*), 60 °C, 2 h	10.21	[27]
High voltage electrical discharges-assisted extraction	Water/sample 20:10 (*v*/*w*), 50 °C, 200 kJ/kg 0,5 Hz	7.00	[6]
Ultrasound-assisted extraction (ultrasonic bath)	Ethanol 40%/sample 80:1 (*v*/*w*), 40 °C, 30 min, 150 W	2.33	[29]
	Ethanol 50%/sample 57:1 (*v*/*w*), 30 °C, 15 min	5.50	[3]
	Ethanol 50%/sample 10:1 (*v*/*w*), 30 min, 400 W	1.05	[28]
	Acetone 75.79%/sample 50:1 (*v*/*w*), 27 °C, 8.33 min, 65.94% amplitude	10.35	[27]
Microwave-assisted extraction	Acetone 51%/sample 25:1 (*v*/*w*), 122 s, 500 W	12.20	[27]
Pressurized liquid extraction	Ethanol 50%/sample 47:1 (*v*/*w*), 10 MPa, 65 °C, 40 min	10.30	[3]
Ultrasound-assisted extraction (ultrasonic bath) combined with enzymatic process	Ethanol/sample 10:1 (*v*/*w*), 25 °C, 120 min, 500 W, with pectinase 0.50% *w*/*w* at pH 4	0.88	[30]
Pressing extraction with pulsed electric fields as pre-treatment	7 kV/cm PEF and 5 bars, 30 min	0.35	[31]
Solvent extraction with infrared as pre-treatment	Ethanol 50%/sample 8:1 (*v*/*w*), 50 °C, 1.5 h	1.5	[32]

**Table 5:** Standard analytes used for elaborating the calibration curves with the range used, equations, *R*^2^, LOD, and LOQ of each compound.

Standards	Calibration Ranges (µg/mL)	Calibration Curves (µg/mL)	*R* ^2^	LOD (µg/mL)	LOQ (µg/mL)
Vanillic acid	3.7–236.67	*y* = 21.069*x* + 197.15	0.9979	0.47	1.57
Chlorogenic acid	3.85–246.67	*y* = 58.665*x* − 289.54	0.9984	0.17	0.56
Ferulic acid	3.54–226.67	*y* = 37.071*x* + 155.61	0.9983	0.27	0.89
Quercetin	3.54–226.67	*y* = 154.26*x* + 1309.1	0.9988	0.06	0.21
Rutin	3.44–220	*y* = 239.6*x* + 690.3	0.9954	0.04	0.14

**Table 6:** Compounds identified by HPLC-ESI-TOF-MS in the optimal extract of orange peel.

No.	Compound	Retention Time (Min)	Molecular Formula	*m/z* Experimental	*m/z* Calculated	Score	Error (ppm)
1	Caffeoylglycolic acid methyl ester isomer a	3.608	C_12_H_12_O_6_	251.055	251.0556	86.53	−2.4
2	Norbergenin	3.806	C_13_H_14_O_9_	313.0563	313.056	94.08	1
3	Citric acid	4.278	C_6_H_8_O_7_	191.0198	191.0192	94.52	3.1
4	Cynaroside A	4.774	C_21_H_32_O_10_	443.191	443.1917	99.27	−1.6
5	2-(E)-*O*-Feruloyl-D-galactaric acid isomer a	4.898	C_16_H_18_O_11_	385.0769	385.0771	99.77	−0.5
6	2-(E)-*O*-Feruloyl-D-galactaric acid isomer b	5.113	C_16_H_18_O_11_	385.0771	385.0771	95.25	0
7	Caffeic acid 3-*O*-glucuronide	5.308	C_15_H_16_O_10_	355.0667	355.0665	99.92	0.6
8	Citroside	5.669	C_19_H_30_O_8_	385.1856	385.1862	87.14	−1.6
9	Ferulic acid *O*-glucoside	5.854	C_16_H_20_O_9_	355.1024	355.1029	97.78	−1.4
10	2-(E)-*O*-Feruloyl-d-galactaric acid isomer c	6.007	C_16_H_18_O_11_	385.0768	385.0771	96.52	−0.8
11	Feruloyl isocitric acid isomer a	6.131	C_16_H_16_O_10_	367.0662	367.0665	99.75	−0.8
12	Sinapic acid *O*-glucoside	6.206	C_17_H_22_O_10_	385.1133	385.1136	99.70	−0.5
13	Caffeoylglycolic acid methyl ester isomer b	6.268	C_12_H_12_O_6_	251.0549	251.0556	88.53	−2.8
14	Caffeoylmalic acid isomer a	6.45	C_13_H_12_O_8_	295.0446	295.0454	98.46	−2.7
15	Sinapinic acid-*O*-glucuronide	6.545	C_17_H_20_O_11_	399.0921	399.0927	89.58	−1.5
16	Feruloyl isocitric acid isomer b	6.661	C_16_H_16_O_10_	367.0675	367.0665	90.04	2.7
17	Rutin isomer a	6.785	C_27_H_30_O_16_	609.1467	609.1456	94.51	1.8
18	Dihydroisorhamnetin 7-rutinoside	7.194	C_28_H_34_O_16_	625.1765	625.1769	87.24	−0.6
19	Apigenin-di-C-hexoside (Vicenin-2) isomer a	7.376	C_27_H_30_O_15_	593.1506	593.1506	99.24	0
20	Prunin	7.596	C_21_H_22_O_10_	433.1132	433.1135	100.0	−0.7
21	Isorhamnetin-3-*O*-rutinoside isomer a	7.753	C_28_H_32_O_16_	623.1607	623.1612	96.55	−0.8
22	Isorhamnetin-3-*O*-rutinoside isomer b	7.914	C_28_H_32_O_16_	623.1605	623.1612	95.79	−1.1
23	Caffeoylmalic acid isomer b	8.241	C_13_H_12_O_8_	295.0428	295.0454	88.92	−8.8
24	Luteolin-*C*-hexoside-*C*-pentoside isomer a	8.432	C_26_H_28_O_15_	579.134	579.135	86.57	−1.7
25	Alpha-Glucosyl Hesperidin	8.481	C_34_H_44_O_20_	771.2352	771.2348	92.05	0.5
26	Luteolin-*C*-hexoside-*C*-pentoside isomer b	8.572	C_26_H_28_O_15_	579.1367	579.135	91.83	2.9
27	Quercitrin	8.676	C_21_H_20_O_11_	447.0934	447.0927	99.92	1.6
28	Eriocitrin	8.8	C_27_H_32_O_15_	595.1662	595.1663	88.90	−0.2
29	Limonin 17-*β*-d-glucopyranoside	8.899	C_32_H_42_O_14_	649.2497	649.2496	99.94	0.2
30	Vitexin-*O*-pentoside isomer a	9.135	C_26_H_28_O_14_	563.1407	563.1401	99.96	1.1
31	Apigenin-di-*C*-hexoside (Vicenin-2) isomer b	9.213	C_27_H_30_O_15_	593.1500	593.1506	96.70	−1
32	Quercetin-*O*-dihexoside	9.234	C_27_H_30_O_17_	625.1411	625.1405	98.61	1
33	Vitexin-*O*-pentoside isomer b	9.511	C_26_H_28_O_14_	563.14	563.1401	100.0	−0.2
34	Naringin hydrate	9.615	C_27_H_34_O_15_	597.1816	597.1819	99.99	−0.5
35	Apigenin-di-*C*-hexoside (Vicenin-2) isomer c	9.697	C_27_H_30_O_15_	593.1506	593.1506	99.13	0
36	Apigenin-di-*C*-hexoside (Vicenin-2) isomer d	9.847	C_27_H_30_O_15_	593.1505	593.1506	98.19	−0.2
37	Narirutin	10.012	C_27_H_32_O_14_	579.1696	579.1714	99.80	0.3
38	Unknown flavonoid	10.074	C_26_H_26_O_14_	561.1246	561.1244	99.56	0.4
39	6-keto-7-*β*-deacetylnomilol 17-*O*-*β*-d-glucopyranoside	10.219	C_32_H_44_O_15_	667.2588	667.2602	88.13	−2.1
40	Rutin isomer b	10.318	C_27_H_30_O_16_	609.1458	609.1456	99.99	0.3
41	Deacetylnomilinic acid 17-*β*-d-glucoside	10.359	C_32_H_46_O_15_	669.2781	669.2758	98.18	3.4
42	Hesperidin	10.45	C_28_H_34_O_15_	609.1812	609.1819	99.98	−1.1
43	Kaempferol 3-*O*-[3″,6″-di-*O*-(E)-cinnamoyl]-*β*-d-glucopyranoside	10.624	C_39_H_32_O_13_	707.1788	707.1765	98.71	3.3
44	Isoobacunoic acid 17-*β*-d-glucoside	10.827	C_32_H_44_O_14_	651.2661	651.2653	98.74	1.2
45	Kaempferol 3-[2″-glucosyl-6″-acetyl-galactoside] 7-glucoside isomer a	10.951	C_35_H_42_O_22_	813.2104	813.2089	99.92	1.8
46	Kaempferol 3-[2″-glucosyl-6″-acetyl-galactoside] 7-glucoside isomer b	11.063	C_35_H_42_O_22_	813.2089	813.2089	97.06	0.2
47	Apigenin-di-*C*-hexoside (Vicenin-2) isomer e	11.208	C_27_H_30_O_15_	593.1504	593.1506	99.92	−0.3
48	Isorhamnetin-3-*O*-rutinoside isomer c	11.336	C_28_H_32_O_16_	623.1614	623.1612	99.20	0.3
49	Kaempferol-dihexosyl acetate	11.613	C_29_H_32_O_17_	651.1577	651.1571	86.35	2.5
50	nomilin 17-*O*-*β*-d-glucopyranoside	11.973	C_34_H_46_O_15_	693.2766	693.2758	99.99	1.2
51	Neohesperidin	12.184	C_28_H_34_O_15_	609.1824	609.1819	99.90	0.8
52	Didymin	12.41	C_28_H_34_O_14_	593.1885	593.187	91.36	2.5
53	Nomilinic acid 17-*β*-d-glucoside	12.602	C_34_H_48_O_16_	711.2878	711.2864	97.47	2
54	Apigenin 7-*O*-neohesperidoside	12.693	C_27_H_30_O_14_	577.1564	577.1557	99.80	1.2
55	Obacunone 17-*β*-d-glucoside	12.742	C_32_H_42_O_13_	633.2574	633.2547	87.20	4.3
56	Naringin 6″-malonate	13.094	C_30_H_34_O_17_	665.1697	665.1718	90.41	−3.2
57	Kaempferol 3-*O*-(6″-*O*-acetyl) glucoside-7-*O*-rhamnoside	13.309	C_29_H_32_O_16_	635.1617	635.1612	99.28	0.8
58	Kaempferol 3-apiosyl-(1->4)-rhamnoside-7-rhamnoside	13.338	C_32_H_38_O_18_	709.1987	709.198	91.26	1
59	Quercetin-3-*O*-rutinoside-7-*O*-Glucoside	13.814	C_33_H_40_O_21_	771.2137	771.1984	89.33	1.8
60	Demethylnobiletin	13.888	C_20_H_20_O_8_	387.1079	387.108	92.26	−0.3
61	Methyl 2-[(2S,4R,5S,6R)-4-acetyloxy-6-(acetyloxymethyl)-5-[(2R,4R,5S,6R)-4,5-diacetyloxy-6-(acetyloxymethyl) oxan-2-yl]oxyoxan-2-yl]oxy-3,4,5-trihydroxy-6-oxobenzo[7]annulene-8-carboxylate	14.132	C_35_H_40_O_20_	779.2047	779.2035	99.98	1.5
62	Naringenin	14.178	C_15_H_12_O_5_	271.0603	271.0606	95.40	−1.1
63	Kaempferol 3-*O*-sinapoyl-caffeoyl-sophoroside 7-*O*-glucoside isomer a	14.339	C_53_H_56_O_28_	1139.2892	1139.2885	95.98	1.1
64	Kaempferol 3-*O*-sinapoyl-caffeoyl-sophoroside 7-*O*-glucoside isomer b	14.476	C_53_H_56_O_28_	1139.2933	1139.288	88.40	4.7
65	Deacetylnomilin acid	14.526	C_26_H_34_O_9_	489.2131	489.2125	90.73	1.2
66	Limonol	14.559	C_26_H_32_O_8_	471.2025	471.2019	96.48	1.3
67	Kaempferol 3-*O*-feruloyl-caffeoyl-sophoroside 7-*O*-glucoside	14.786	C_52_H_54_O_27_	1109.2789	1109.2774	90.94	1.4
68	Epilimonin	15.043	C_26_H_30_O_8_	469.1859	469.1862	99.98	−0.6
69	Limonin	15.708	C_26_H_30_O_8_	469.1849	469.1862	99.28	−2.8
70	Nomilinic acid	15.8	C_28_H_36_O_10_	531.2208	531.223	91.17	−4.1
71	Pectolinarigenin	16.073	C_17_H_14_O_6_	313.0704	313.0712	98.95	−2.6
72	Deoxylimonin	16.102	C_26_H_30_O_7_	453.1881	453.1913	85.92	−3.1
73	Isosakuranetin	16.524	C_16_H_14_O_5_	285.0754	285.0763	89.61	−3.2
74	3′,4′-Didemethylnobiletin	16.569	C_19_H_18_O_8_	373.0923	373.0923	94.17	0

**Table 7:** Quantification of phenolic compounds from orange peel dry and fresh by HPLC-MS (expressed as mean ± standard deviation µg/g d.w.).

Compounds	Fresh By-Product (µg/g d.w.)	Dry By-Product (µg/g d.w.)
Sum of phenolic compounds	6344.0 ± 3.6	4737.6 ± 4.4
Phenolic Acids	3087.5 ± 0.2	2891.1 ± 2.4
Norbergenin	383.5 ± 0.5	340.4 ±0.7
Caffeoylglycolic acid methyl ester isomer a	201.8 ± 0.1	284.2 ± 0.7
Caffeoylglycolic acid methyl ester isomer b	133.36 ± 0.04	178.2 ± 0.1
Caffeic acid 3-*O*-glucuronide	218.9 ± 0.3	128.6 ± 0.7
Caffeoylmalic Acid isomer a	127.9 ± 0.6	99.5 ± 0.4
Caffeoylmalic Acid isomer b	109.5 ± 0.1	77.6 ± 0.1
2-(E)-*O*-Feruloyl-D-galactaric acid isomer a	378.8 ± 0.5	463.0 ± 0.04
2-(E)-*O*-Feruloyl-D-galactaric acid isomer b	575.6 ± 0.3	416.3 ± 0.4
2-(E)-*O*-Feruloyl-D-galactaric acid isomer c	313.2 ± 0.2	564.0 ± 0.9
Ferulic acid *O*-glucoside	235.3 ± 0.7	48.73 ± 0.1
Feruloyl Isocitric acid isomer a	195.2 ± 0.7	170.66 ± 0.4
Feruloyl isocitric acid isomer b	16.9 ± 0.03	11.2 ± 0.2
Sinapic acid *O*-glucoside	183.5 ± 0.4	14.9 ± 0.1
Sinapinic acid-*O*-glucuronide	14.0 ± 0.5	93.7 ± 0.1
Flavonoids	3256.5 ± 3.4	1846.5 ± 2.0
Cynaroside A	13.1 ± 0.3	6.1 ± 0.1
Rutin isomer a	57.8 ± 0.3	73.6 ± 0.1
Rutin isomer b	22.0 ± 0.1	49.8 ± 0.05
Prunin	122.5 ± 0.4	35.4 ± 0.01
Quercitrin	1.2 ± 0.2	<LOQ
Eriocitrin	24.6 ± 0.1	17.5 ± 0.4
Narirutin	799.4 ± 0.5	319.6 ± 0.4
Hesperidin	894.8 ± 0.5	320.2 ± 0.5
α-glucosyl Hesperidin	23.8 ± 0.1	19.0 ± 0.1
Neohesperidin	<LOQ	<LOQ
Didymin	146.3 ± 0.2	72.8 ± 0.2
Naringin 6″-malonate	<LOQ	<LOQ
Naringin hydrate	35.1 ± 0.2	51.7 ± 0.2
Dihydroisorhamnetin 7-rutinoside	8.4 ± 0.2	10.1 ± 0.06
Isorhamnetin-3-*O*-rutinoside isomer a	135.4 ± 0.6	116.6 ± 0.3
Isorhamnetin-3-*O*-rutinoside isomer b	10.6 ± 0.1	8.0 ± 0.0005
Isorhamnetin-3-*O*-rutinoside isomer c	54.6 ± 0.1	48.9 ± 0.002
Vitexin-*O*-pentoside isomer a	72.2 ± 0.5	68.61 ± 0.27
Vitexin-*O*-pentoside isomer b	111.5 ± 0.01	94.6 ± 0.2
Apigenin 7-*O*-neohesperidoside	14.8 ± 0.1	9.2 ± 0.5
Apigenin-di-*C*-hexoside (Vicenin-2) isomer a	570.7 ± 0.9	318.3 ± 0.4
Apigenin-di-*C*-hexoside (Vicenin-2) isomer b	<LOQ	<LOQ
Apigenin-di-*C*-hexoside (Vicenin-2) isomer c	7.7 ± 0.2	13.7 ± 0.1
Apigenin-di-*C*-hexoside (Vicenin-2) isomer d	22.3 ± 0.1	16.3 ± 0.1
Apigenin-di-*C*-hexoside (Vicenin-2) isomer e	<LOQ	<LOQ
Luteolin-*C*-hexoside-*C*-pentoside isomer a	1.2 ± 0.1	0.2 ± 0.01
Luteolin-*C*-hexoside-*C*-pentoside isomer b	3.8 ± 0.1	2.3 ± 0.02
Kaempferol 3-[2″-glucosyl-6″-acetyl-galactoside] 7-glucoside isomer a	24.6 ± 0.3	16.0 ± 0.004
Kaempferol 3-[2″-glucosyl-6″-acetyl-galactoside] 7-glucoside isomer b	16.9 ± 0.7	12.3 ± 0.04
Kaempferol-dihexosyl acetate	<LOQ	<LOQ
Kaempferol 3-*O*-(6″-*O*-acetyl) glucoside-7-*O*-rhamnoside	<LOQ	<LOQ
Kaempferol 3-apiosyl-(1->4)-rhamnoside-7-rhamnoside	<LOQ	<LOQ
kaempferol 3-*O*-[3″,6″-di-*O*-(E)-cinnamoyl]-*β*-d-glucopyranoside	0.7 ± 0.06	0.3 ± 0.002
Kaempferol 3-*O*-sinapoyl-caffeoyl-sophoroside 7-*O*-glucoside isomer a	<LOQ	<LOQ
Kaempferol 3-*O*-sinapoyl-caffeoyl-sophoroside 7-*O*-glucoside isomer b	<LOQ	<LOQ
Kaempferol 3-*O*-feruloyl-caffeoyl-sophoroside 7-*O*-glucoside isomer c	<LOQ	<LOQ
Quercetin-*O*-dihexoside	<LOQ	<LOQ
Quercetin-3-*O*-rutinoside-7-*O*-Glucoside	<LOQ	<LOQ
Demethylnobiletin	n.d.	<LOQ
3′,4′-Didemethylnobiletin	<LOQ	<LOQ
Naringenin	11.5 ± 0.02	9.4 ± 0.009
Pectolinarigenin	<LOQ	<LOQ
Isosakuranetin	n.d.	<LOQ
Unknown flavonoid	60.3 ± 0.1	125.8 ± 0.5

n.d.: non-detected; LOQ: Limit of quantification.

## Data Availability

Not applicable.
